# Effect of temperature and large guest molecules on the C–H symmetric stretching vibrational frequencies of methane in structure H and I clathrate hydrates†

**DOI:** 10.1039/d0ra02748k

**Published:** 2020-05-06

**Authors:** Go Fuseya, Satoshi Takeya, Akihiro Hachikubo

**Affiliations:** Kitami Institute of Technology 165, Koen-cho Kitami 090-8507 Japan hachi@mail.kitami-it.ac.jp; National Institute of Advanced Industrial Science and Technology (AIST) Central 5, 1-1-1, Higashi Tsukuba 305-8565 Japan

## Abstract

Large molecules such as 2-methylbutane (C_5_H_12_) or 2,2-dimethylbutane (C_6_H_14_) form structure H (sH) hydrates with methane (CH_4_) as a help gas. In this study, the Raman spectra of the C–H symmetric stretch region of CH_4_ enclathrated within various sH hydrates and structure I CH_4_ hydrates were analyzed in the temperature range 137.7–205.4 K. Thermal expansions of these sH hydrate samples were also measured using powder X-ray diffraction. Symmetric stretch vibrational frequencies of CH_4_ in host–water cages increased because of varying temperature, and the sizes of the host–water cages also increased; variation of CH_4_ in small cages was less than in larger cages. Comparing the variations of the C–H symmetric stretching frequencies of CH_4_ in gas hydrates with varying pressure and temperature, we suggest that the observed trend is caused by thermal vibrations of the CH_4_ molecule in water cages.

## Abstract 2

Temperature effect on C–H symmetric stretching frequencies of CH_4_ in water cages of sI and sH clathrate hydrates were clarified.

## Graphical abstract

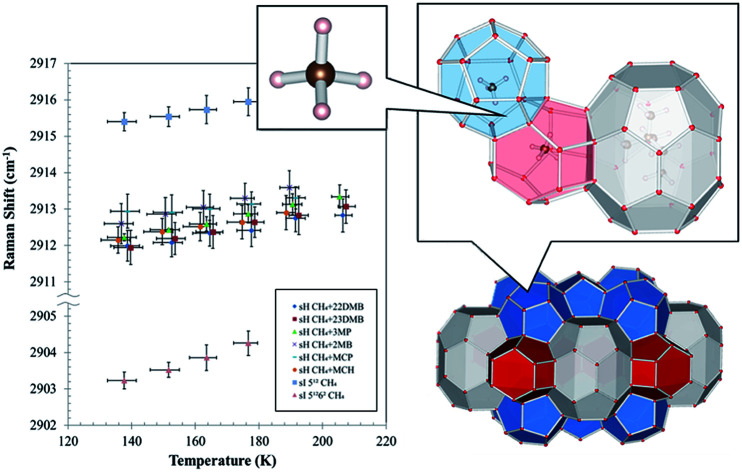

## Introduction

Clathrate hydrates are crystalline inclusion compounds that consist of guest molecules of suitable sizes and shapes trapped in well-defined cages formed by water molecules. Both synthetic and naturally occurring clathrate hydrates with natural gases as guest molecules are commonly known as gas hydrates. Gas hydrates with enclathrated hydrocarbon gases that exist in sea/lake bottom sediments and permafrost layers have attracted considerable interest as a potential source of unconventional natural gas.^1^

**Fig. 1:**
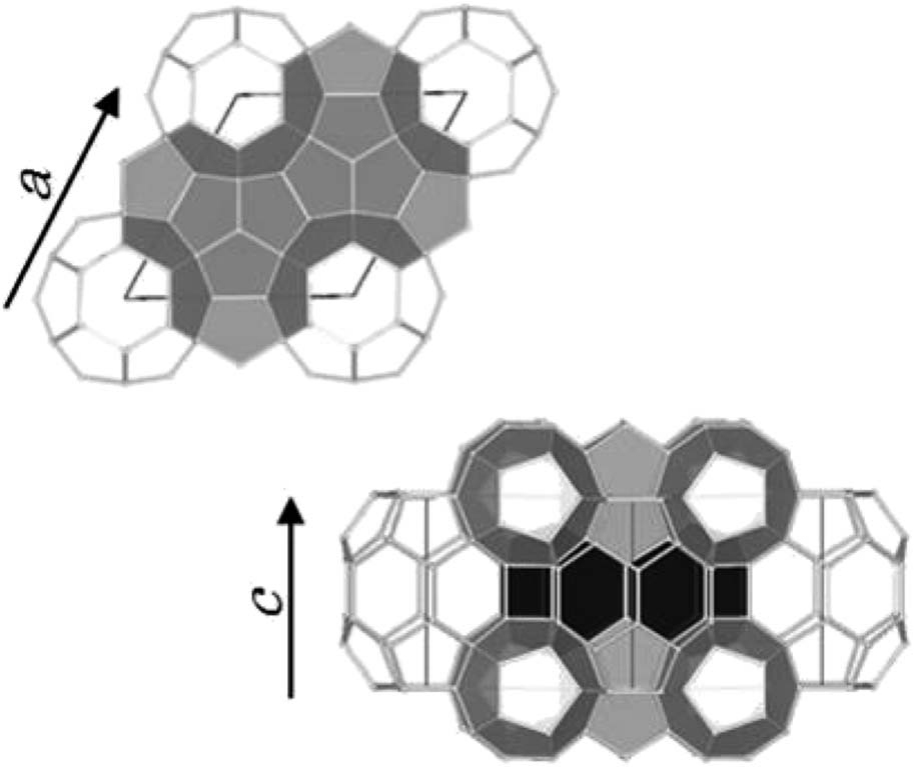
Unit cell of sH hydrates. The unit-cell comprises three 5^12^ cages (light gray), two 4^3^5^6^6^3^ cages (black), and one 5^12^6^8^ cage (white).

Three typical crystal structures of clathrate hydrates have been identified on earth: cubic structure I (sI), cubic structure II (sII), and hexagonal structure H (sH).^2–4^ The unit cell of sI hydrates comprises two pentagonal dodecahedral (5^12^) and six tetrakaidecahedral (5^12^6^2^) water cages.^2^ For sII hydrates, the unit cell is formed by sixteen 5^12^ cages and eight hexakaidecahedral (5^12^6^4^) water cages.^3^ Finally, the unit cell of sH hydrates comprises three 5^12^ cages, two irregular dodecahedral (4^3^5^6^6^3^) cages, and one icosahedral (5^12^6^8^) cage^4^ as shown in Fig. 1. Small guest molecules such as methane (CH_4_) or ethane form sI-type hydrates, whereas larger molecules like propane or 2-methylpropane (C_4_H_10_) form sII hydrates. sH hydrates are obtained from even larger molecules, *e.g.*, 2-methylbutane (C_5_H_12_) or 2,2-dimethylbutane (C_6_H_14_), in the presence of a help gas as CH_4_.^1^ Moreover, sH hydrate that is capable of encapsulating these larger molecules in natural gas than either structure I or II hydrates was found at the Barkley Canyon (northern Cascadia margin).^5^ Since sH hydrates exhibit a hexagonal structure (space group *P*6/*mmm*), there are two unit-cell parameters, *a*-axis and *c*-axis (Fig. 1). Under isothermal conditions, the lattice size of clathrate hydrates has been reported to change depending on the type of guest molecule. For sH hydrates, large guests cause an increase in the *a*-axis direction and a slight decrease in the *c*-axis direction.^6^

To date, Raman spectroscopy has been commonly used for gas hydrates to identify the type of crystal phase^7–9^ and phase transformations^10,11^ or to analyze cage occupancy,^7^ type of guest molecule,^9^ and hydration number.^7,12^ The Raman spectra of the C–H symmetric stretch region of enclathrated CH_4_ are commonly used to identify the types of crystal phase and guest molecules.^13^ Trends in the C–H stretching vibrational frequency of CH_4_ enclosed in different clathrate hydrate cages have therefore been studied. Experimentally, the Raman spectra of hydrocarbon hydrates have demonstrated that the C–H stretching frequency of CH_4_ in large cages is generally lower than that for CH_4_ in small cages.^7–9^ This observation was rationalized by Subramanian and Sloan^9^ in terms of the guest–cage intermolecular interactions using the loose cage–tight cage (LCTC) model as an explanation for matrix-isolation IR experiments.^14^ Also, a qualitative agreement between experimental results and theoretical calculations for CH_4_ enclathrated in sI and sH hydrates was obtained using *ab initio* MD simulations.^15–17^ Additionally, using quantum-chemical computations, the C–H stretching frequencies of hydrocarbons in different clathrates were demonstrated to be lower in larger cages.^18^ This vibrational frequency for CH_4_ enclosed in a water cage may change depending on temperature, and these information are useful for better understanding of the stability of gas hydrates under natural setting. However, thermal effects such as thermal expansion of water cages have not been considered for these calculations yet.

In this study, various sH CH_4_ and large-molecule mixed hydrates and an sI CH_4_ hydrate were prepared, and the Raman spectra in the C–H symmetric stretch region of enclathrated CH_4_ were studied in a temperature range of 137.7–205.4 K. Thermal expansions of all samples were also measured using powder X-ray diffraction (PXRD). From these results, the variations in Raman spectra of the C–H symmetric stretch and the guest–host interaction energy with varying temperature are discussed; moreover, the differences in crystal structures are analyzed. The results of this study help to consider computational elements of follow theoretical calculation study of thermal effect on sH hydrate and sI CH_4_ hydrates, also may help to better understand the effect of temperature on clathrate hydrate stability and guest–host interactions.

## Experimental section

### Sample preparation

**Fig. 2:**
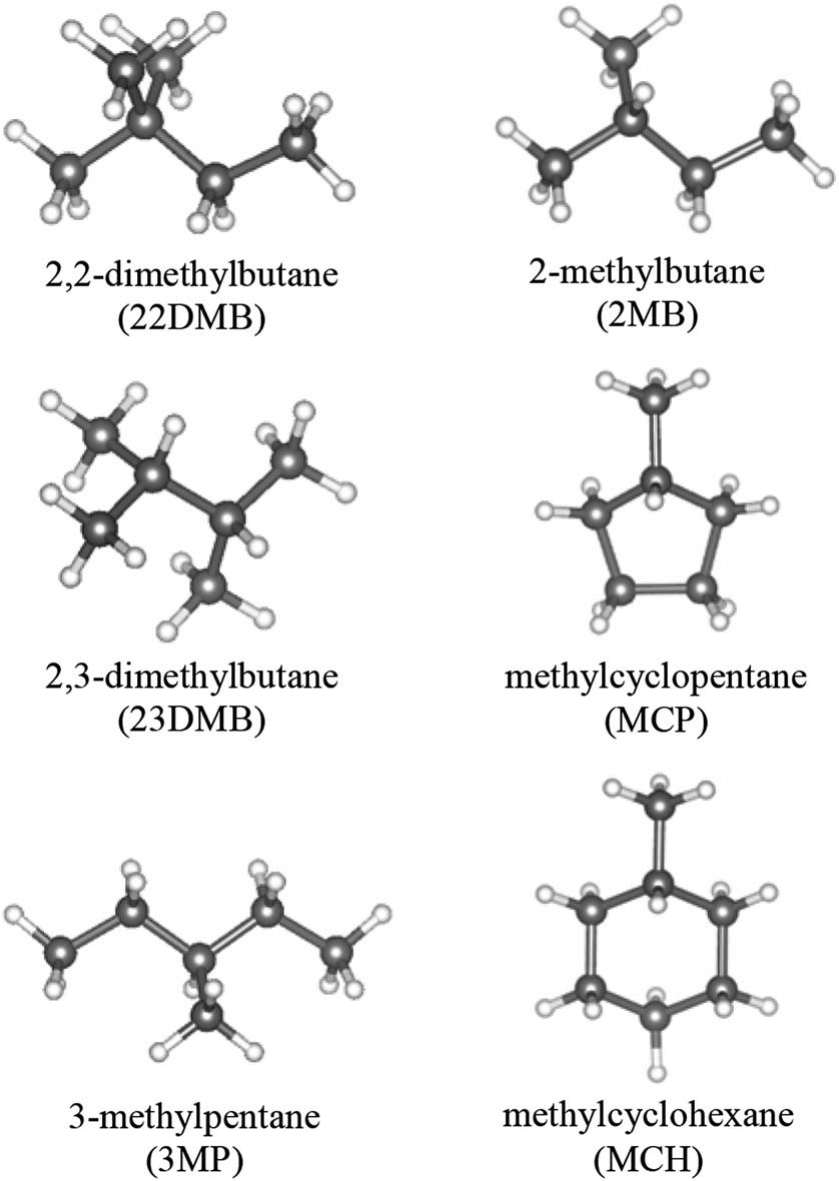
Structures of the large-molecule guest.

In this study, six large-molecule guest substances as shown in Fig. 2 were used. Six gas hydrate samples were prepared: CH_4_ + 2,2-dimethylbutane (22DMB); CH_4_ + 2,3-dimethylbutane (23DMB); CH_4_ + 3-methylpentane (3MP); CH_4_ + 2-methylbutane (2MB); CH_4_ + methylcyclopentane (MCP); and CH_4_ + methylcyclohexane (MCH). To synthesize these gas hydrate samples, fine ice powder (3.0 g) was prepared and loaded onto a high-pressure cell (internal volume: ∼20 mL), which was precooled in a freezer at 253 K. Afterwards, 0.3 mL of the large-molecule guest was added into the cell (Wako Pure Chemical Industries Ltd.; 22DMB 99.3%; 23DMB 99.9%; 3MP 99.6%; 2MB 99.6%; MCP 97.9%; MCH 99.8%). To prevent melting of ice or evaporation of the guest molecules, the high-pressure cell and hydrate components were precooled in a cold room at 253 K. After loading at 253 K, the high-pressure cell was cooled to below 90 K and pure CH_4_ gas (99.99% certified purity; Takachiho Chemical Industrial Co. Ltd.) was slowly introduced into the cell. Then, the high-pressure cell was transferred into a water bath at 273.2 K for hydrate formation. The CH_4_ applied pressure was maintained at 2.2–2.3 MPa to not form pure sI CH_4_ hydrate. As the hydrates formed, the pressure decreased. When the pressure stabilized, the cell was cooled below 90 K and the sample was removed from the cell. For the sI CH_4_ hydrate (MH), powder ice (3.0 g) was loaded into the cell, and the cell was pressurized using CH_4_ to over 2.6 MPa.

### Raman spectroscopy

A Raman spectrometer (JASCO Corporation, RMP-210) equipped with a 532 nm excitation source (100 mW), a single holographic diffraction grating (1800 grooves per mm), and a CCD detector was used. The spectrum pixel resolution, which is the sampling interval of the spectrum, was 0.9 cm^−1^ per pixel in the range of 2500–3000 cm^−1^. A polypropylene peak at 1460 cm^−1^ was used for routine calibration of the monochromator. The wave number was also calibrated using atomic emission lines from a neon lamp. The Raman spectra for the C–H symmetric stretch region of enclathrated CH_4_ in small cages of six sH mixed-gas hydrates and the sI CH_4_ hydrate were obtained for a temperature range of 137.7–205.4 K. The sample temperature was confirmed using a thermocouple (Type T; Ninomiya Electric Wire Co. Ltd., 01-T). The calibrated thermocouple was accurate within 0.1 °C. Here, the peak position can be rigorously analysed by Voigt fitting functions to obtain a high positional accuracy. Based on the reproducibility of the CH_4_ peak position of the sH 5^12^ and 4^3^5^6^6^3^ cages of CH_4_ + 2MB hydrate at 163.6 K, the standard deviation of peak positions, which is 18 times measurement at same sample position, was approximately 0.1 cm^−1^.

### Powder X-ray diffraction

PXRD measurements were performed using a laboratory X-ray diffractometer (40 kV, 40 mA; RIGAKU model Ultima-III) with parallel beam optics and a low-temperature chamber. Fine powder hydrate samples were mounted on a PXRD sample holder made of 2.5 mm thick Cu at a temperature comparable to liquid N_2_ (∼100 K). Each measurement was obtained in a *θ*/2*θ* step scan mode with a step width of 0.02° using Cu Kα radiation (*λ* = 1.541 Å).

## Results and discussion

**Fig. 3:**
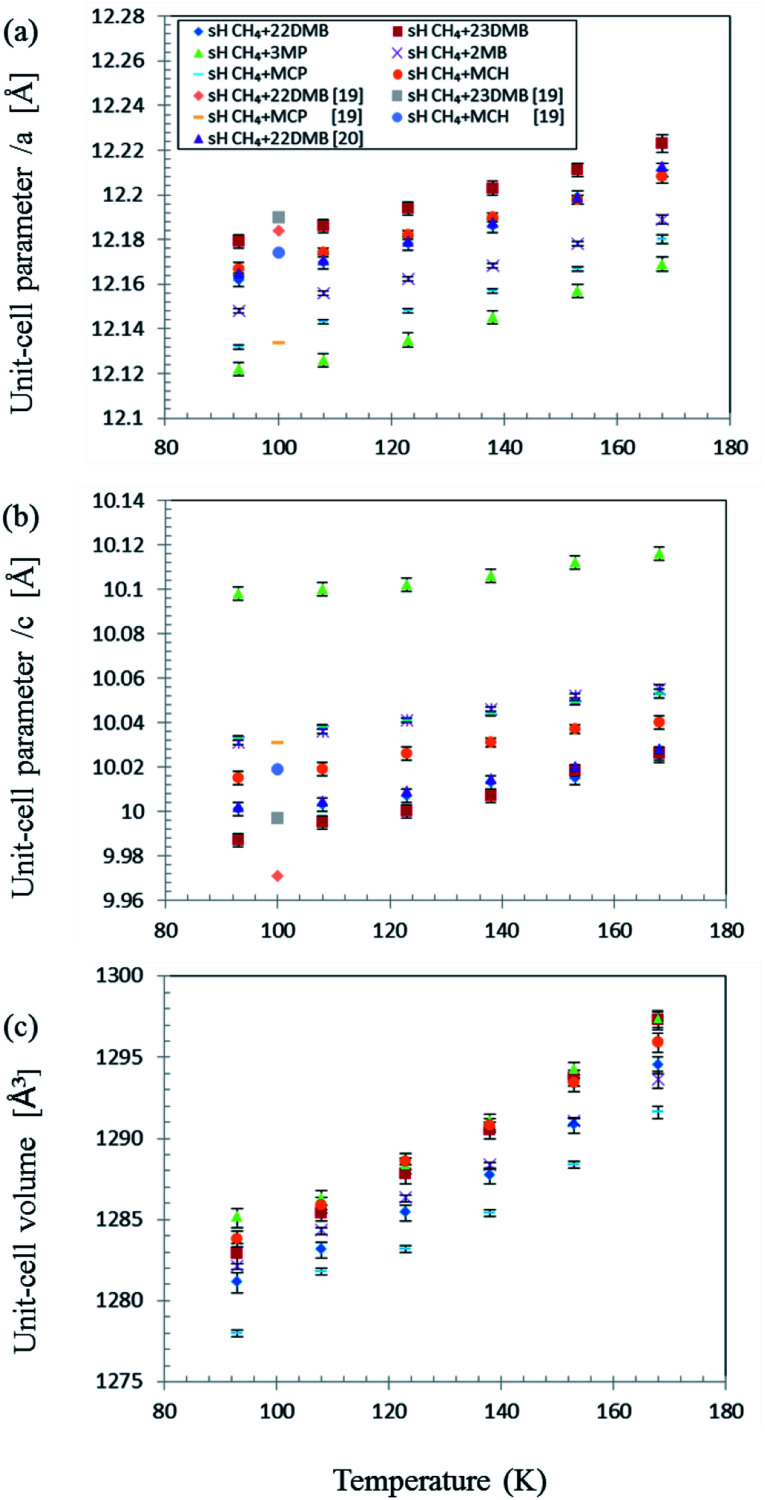
Effect of temperature on sH hydrate unit-cell parameters with various guest molecules and CH_4_. (a) *a*-axis; (b) *c*-axis; (c) unit-cell volume.^19,20^

As expected, PXRD measurements showed that the crystal structure of the six different gas hydrates was sH (Fig. S1†). The effect of temperature on the unit-cell parameters in the range of 93–168 K is shown in Fig. 3 for all samples. Each unit-cell size and effect of temperature varied with varying unit-cell parameters differ with guest molecules. However, unit-cell parameters of all the samples increased with increasing temperature and volume expansion of unit cells.

**Fig. 4:**
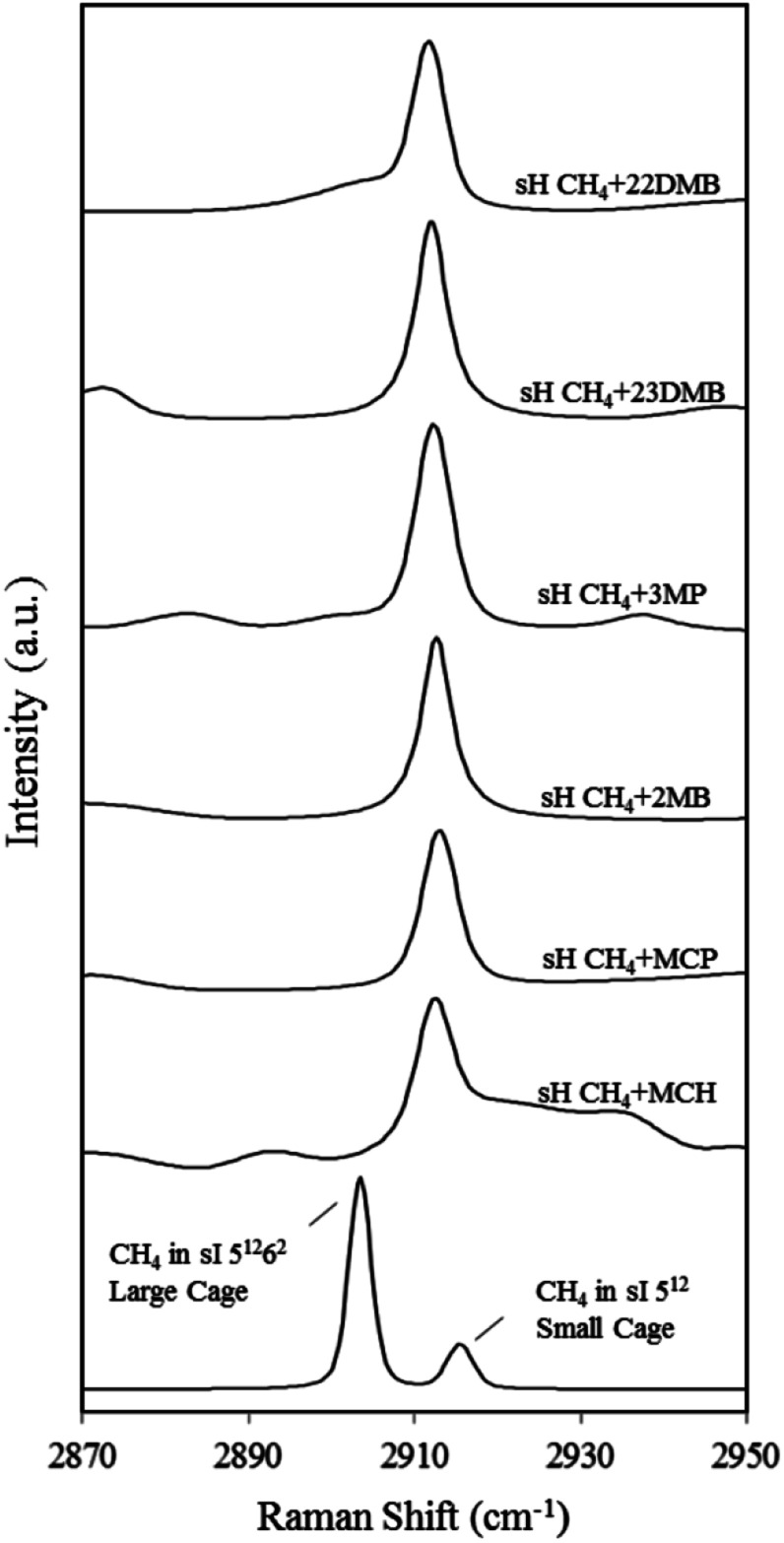
Raman spectra of CH_4_ in the 5^12^ (small) and 5^12^6^2^ (large) cages of the sI hydrate (MH) and the 5^12^ and 4^3^5^6^6^3^ cages of six types of sH hydrates at 137.7 K. Raman peaks for CH_4_ in the 5^12^ and 4^3^5^6^6^3^ sH cages overlap; only one peak was observed.

Fig. 4 also depicts the C–H symmetric stretch of CH_4_ in the 5^12^ and 5^12^6^2^ cages of the sI CH_4_ hydrate for comparison. Information from each Raman spectrum of CH_4_ in the 5^12^ and 5^12^6^2^ cages is summarized in Table 1. In the case of sI CH_4_ hydrate, Raman spectra on CH_4_ vibrations in the 5^12^ and 5^12^6^2^ cages agree well with those of natural methane hydrate^21^ and results from molecular dynamics simulation.^22^ Sum *et al.* reported that the Raman peaks of CH_4_ in the 5^12^ cages of sI and sH are 2915 and 2913 cm^−1^, respectively.^7^ Experimental results in this study are in agreement with the earlier study. It is suggested that CH_4_ is enclathrated into the sH 5^12^6^8^ cages under high-pressure condition at above 1.36 GPa and the Raman peak appears around 2930 cm^−1^.^23^ However, such peaks were not detected at around 2930 cm^−1^ (Fig. 4) because large molecules fully occupied the 5^12^6^8^ cages as is reported in earlier studies.^24–26^

**Table 1:** Raman shifts of C–H symmetric stretch CH_4_ in 5^12^ and 5^12^6^2^ cages of sI hydrate, 5^12^ and 4^3^5^6^6^3^ cages of six types sH hydrates and these variations with temperature. Those errors are the standard deviation of 9 times measurement at different sample positions

Cage type and guest molecules	Raman shift of CH_4_ at 137.7 K [cm^−1^]	Slope of Raman shift between 137.7–205.4 K [cm^−1^/100 K]
sH CH_4_ + 22DMB	2912.0 ± 0.2	1.2
sH CH_4_ + 23DMB	2911.9 ± 0.2	1.7
sH CH_4_ + 3MP	2912.2 ± 0.2	1.7
sH CH_4_ + 2MB	2912.6 ± 0.4	1.9
sH CH_4_ + MCP	2912.9 ± 0.3	0.7
sH CH_4_ + MCH	2912.2 ± 0.2	1.4
sI 5^12^ CH_4_	2915.4 ± 0.1	1.5
sI 5^12^6^2^ CH_4_	2903.2 ± 0.2	2.8

**Fig. 5:**
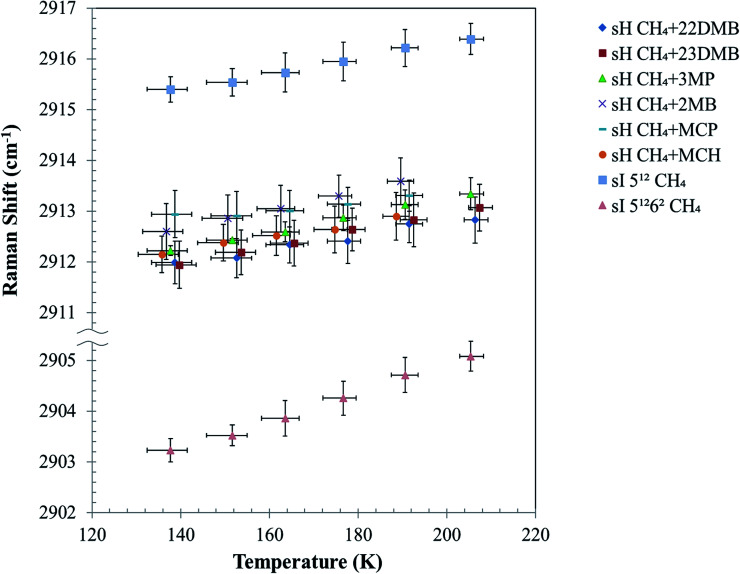
Temperature effect on Raman shift of C–H symmetric stretch of enclathrated CH_4_ in 5^12^ and 4^3^5^6^6^3^ cages of six types sH hydrates and in 5^12^ (small) and 5^12^6^2^ (large) cages of sI CH_4_ hydrate (MH). In the case of CH_4_ + 2MB, CH_4_ + MCP and CH_4_ + MCH, samples dissociated at 205.4 K. The errors in the temperature are the max and minimum values of 10 times measurements.

Fig. 5 depicts the effect of temperature on the C–H symmetric stretch of CH_4_. These Raman shifts increased with increasing temperature. For CH_4_ + 22DMB, CH_4_ + 23DMB, CH_4_ + 3MP, CH_4_ + 2MB, and CH_4_ + MCH hydrates, the slope of Raman shifts was about 1.2–1.9 cm^−1^/100 K, whereas for CH_4_ + MCP, it was 0.7 cm^−1^/100 K (Table 1). This is consistent with the C–H stretching frequency of gaseous CH_4_, which shifts to higher wavenumbers as the temperature increases.^27^ Except for CH_4_ + MCP, the temperature effect on the Raman shift of CH_4_ in the sH hydrate seemed to be the same as that for CH_4_ in the 5^12^ cages of sI CH_4_ hydrates. These results suggest that there is a general trend of the Raman shift with the size of the host cage. If Raman measurements with higher resolution can be obtained, it is possible to discuss effect of temperature depending on the size and/or shape of large guest molecules.

**Fig. 6:**
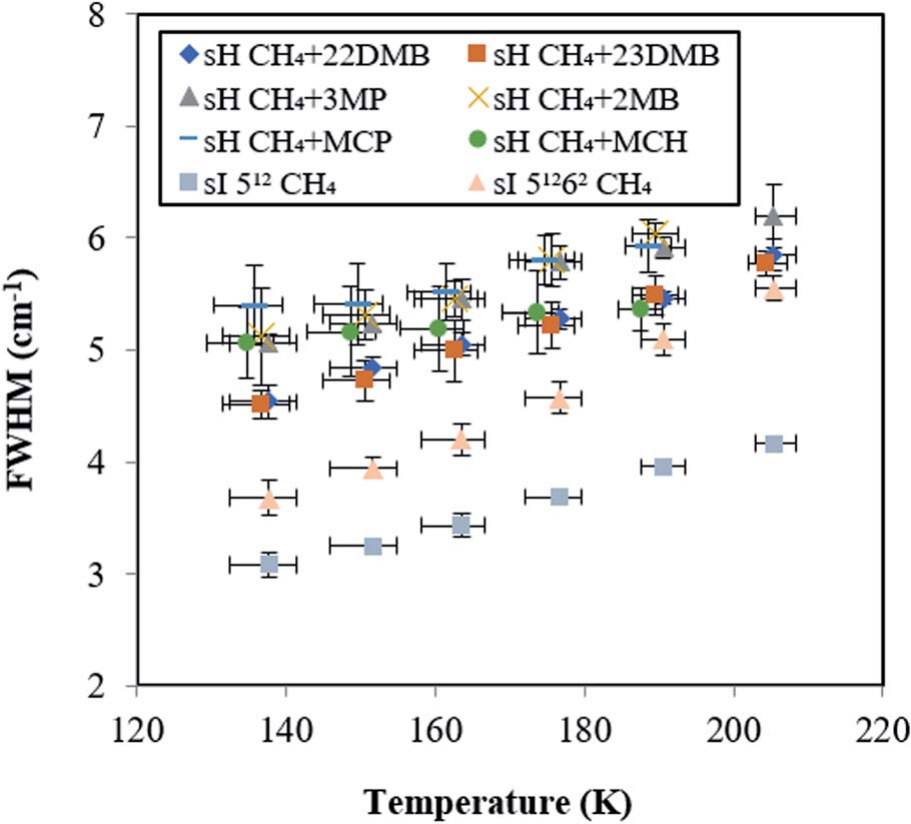
Temperature effect on peak full widths at half maximums (FWHMs) for CH_4_ in sI and in six types sH hydrates. These errors in the FWHM of 0.4 cm^−1^ are standard deviation of 9 times measurement.

To evaluate the increase with temperature in the Raman shift of CH_4_ in the sH hydrate, the overlap of Raman peaks for the 5^12^ and 4^3^5^6^6^3^ cages needs to be considered. The temperature effect on peak full widths at half maxima (FWHMs) of all peaks from Fig. 5 are shown in Fig. 6. FWHMs of all samples increased when temperature increased from 137.7 to 190.6 K. Here, we focus on the values of increase in FWHMs from 137.7 to 190.6 K. In the case of CH_4_ + 22DMB, CH_4_ + 23DMB, CH_4_ + 3MP, CH_4_ + 2MB, and the sI 5^12^ cage, the FWHMs increased by 0.86–0.97 cm^−1^. In the case of CH_4_ + MCP, CH_4_ + MCH, and the sI 5^12^6^2^ cage, the FWHMs increased by 0.53 cm^−1^, 0.30 cm^−1^ and 1.42 cm^−1^, respectively. The variations of the effect of temperature on FWHMs were almost the same or lower for the overlapped sH CH_4_ peaks than those for the sI CH_4_ Raman peaks in the 5^12^ and 5^12^6^2^ cages. Hence, it was indicated that the effects of temperature on the sH CH_4_ Raman peaks in both 5^12^ and 4^3^5^6^6^3^ cages are comparable to each other, although each of the Raman peak for CH_4_ in the sH 5^12^ and 4^3^5^6^6^3^ cages were not measured separately.

The C–H stretching frequency of the guest CH_4_ is usually lower in larger cages than in smaller ones under isothermal conditions, as predicted by the LCTC model.^9,15,18^ The experimental results in this study indicate that Raman shifts of the C–H symmetric stretch of the guest CH_4_ increased with temperature because of thermal vibration of the CH_4_ molecule in water cages; however, cage size also increased, and this might induce a decrease in Raman shifts due to unit-cell expansions with temperature (Fig. 3).

Fig. 5 illustrates that the effect of temperature on Raman shifts of the sI 5^12^, sH 5^12^, and sH 4^3^5^6^6^3^ cages were smaller than that of the sI 5^12^6^2^ cage. This may suggest different interactions between the host, water, and guest (CH_4_) in these cages. In previous studies, the distribution of CD_4_ molecules in a sI deuterated hydrate cage was analyzed using neutron diffraction experiments.^28,29^ It was shown that CD_4_ in the sI 5^12^6^2^ cage distributes longitudinally within the cage at temperatures higher than 80 K, whereas in the 5^12^ cage, CD_4_ distributes spherically around the center of the cage even at higher temperatures. It was suggested that the distance change between the guest and host molecules in the sI 5^12^6^2^ cage is smaller than that in the sI 5^12^ cage due to changes in temperature. Accordingly, the experimental results in Fig. 5 show that the Raman shifts of the C–H symmetric stretch of enclathrated CH_4_ in the sI 5^12^6^2^ cage increased with temperature more than in the sI 5^12^, sH 5^12^, and sH 4^3^5^6^6^3^ cages. The size of these small cages might be too small for translational motion of CH_4_ within the cages.

The CH_4_ hydrate phase is known to transition from the sI to sH structure at 0.9 GPa, and the structure is maintained until about 1.9 GPa.^25^ In this work, the Raman shift of CH_4_ in the sH 5^12^ and 4^3^5^6^6^3^ cages was larger than that for the sI 5^12^ cage at 0.9 GPa.^30^ This suggests that the sH 5^12^ cage is smaller than the sI 5^12^ cage as predicted by the LCTC model.^9,15,18^ Conversely, variations in the C–H symmetric stretching frequency of CH_4_ with changing pressure under isothermal conditions are not the same as the temperature-induced changes obtained in this study. With pressures of up to 0.8 GPa, the variation of the C–H symmetric stretching frequency of CH_4_ in the sI 5^12^ cage is higher than that in the sI 5^12^6^2^ cage.^30^ Contrastingly, the variations of the frequencies of CH_4_ enclathrated in larger cages (sI 5^12^6^2^ and sH 5^12^6^8^ cage) are smaller than that in the 5^12^ and 4^3^5^6^6^3^ cages with changing pressure.^30^ Therefore, it can be implied that the increase in temperature enhances the C–H symmetric stretching frequency of guest CH_4_ because of thermal vibration, whereas the increase in pressure enhances this stretching frequency because of shrinking of cages, as predicted by the LCTC model. The increase in pressure from 0.9 GPa to 1.9 GPa under isothermal conditions causes a volume reduction of 3% for sH,^31^ and the increase in temperature from 93 K to 168 K causes a volume expansion of 1% for sI and sH (Fig. S2†). Therefore, the difference in these volume expansion ratios affects the trend of C–H stretching frequency of CH_4_ in the cages.

These experimental trends of temperature effect of C–H stretching frequency of CH_4_ in the water cages may help to consider computational elements of follow theoretical calculation study of thermal effect on sH hydrate and sI CH_4_ hydrates, although it is not yet complete. For example, the difference in unit-cell sizes is different depending on the type of guest molecule and may change the guest–host interactions. In fact, for CH_4_ + 22DMB, CH_4_ + 23DMB, CH_4_ + 3MP, CH_4_ + 2MB, CH_4_ + MCH; the Raman shifts increased by 0.7–1.0 cm^−1^, but that of CH_4_ + MCP was smaller. Raman measurements with higher resolution coupled with precise crystal structure data are necessary for further understanding of other correlating factors.

## Conclusions

In this work, six different sH clathrate hydrates with CH_4_ as a help gas were investigated: CH_4_ + 22DMB; CH_4_ + 23DMB; CH_4_ + 3MP; CH_4_ + 2MB; CH_4_ + MCP; and CH_4_ + MCH. PXRD measurements and Raman spectra of the C–H symmetric stretch region of the enclathrated CH_4_ within these sH hydrates were analyzed in the temperature range of 137.7–205.4 K.

The C–H symmetric stretch vibrational frequencies of enclathrated CH_4_ in the sH and sI hydrates increase with increasing temperature. Raman shifts of the C–H symmetric stretch vibrational frequencies of CH_4_ enclathrated in larger cages decreased than in smaller cages. However, in this work, these Raman shifts increased even though unit-cell sizes of all samples increased with increasing temperature.

The distribution of CH_4_ in the water cage increases with increasing temperature, implying that the distance between the CH_4_ and water molecules of the lattice is small. The variation of the C–H symmetric stretching frequency of CH_4_ in the large cages was greater than that in small cages, because the thermal vibration of CH_4_ in the former were bigger than that in the latter.

These results contribute to understand the stability and guest–host interactions of sH hydrate and sI CH_4_ hydrates. These results of C–H stretching frequency of CH_4_ in the water cages may help to consider computational elements of follow theoretical calculation study of thermal effect on sH hydrate and sI CH_4_ hydrates. However, the trend of the Raman spectra peaks of CH_4_ in the sH hydrate has not yet been interpreted. For example, variations of the C–H symmetric stretching frequency of CH_4_ in sH hydrates vary depending on the guest molecule. To further comprehend other correlating factors, Raman spectroscopy with higher resolution coupled with precise crystal structure data is needed.

## Conflicts of interest

There are no conflicts to declare.

## Supplementary Material

RA-010-D0RA02748K-s001
