# Supplementary material for: Effect of temperature and large guest molecules on the C–H symmetric stretching vibrational frequencies of methane in structure H and I clathrate hydrates
Source: RSC Adv. 2020 May 6;10(30):17473–8. doi: 10.1039/d0ra02748k (PMC9053386; doi:10.1039/d0ra02748k)
Supplement: RA-010-D0RA02748K-s001 [file RA-010-D0RA02748K-s001.pdf]

Electronic Supplementary Material (ESI) for RSC Advances.
This journal is © The Royal Society of Chemistry 2020

Supporting Information

Effect of Temperature and Large Guest Molecules on the C–H Symmetric Stretching Vibrational Frequencies of Methane in Structure H and I Clathrate Hydrates

Go Fuseya and Akihiro Hachikubo*
Kitami Institute of Technology, 165, Koen-cho, Kitami 090-8507, Japan

Satoshi Takeya
National Institute of Advanced Industrial Science and Technology (AIST), Central 5, 1-1-1, Higashi, Tsukuba 305-8565, Japan

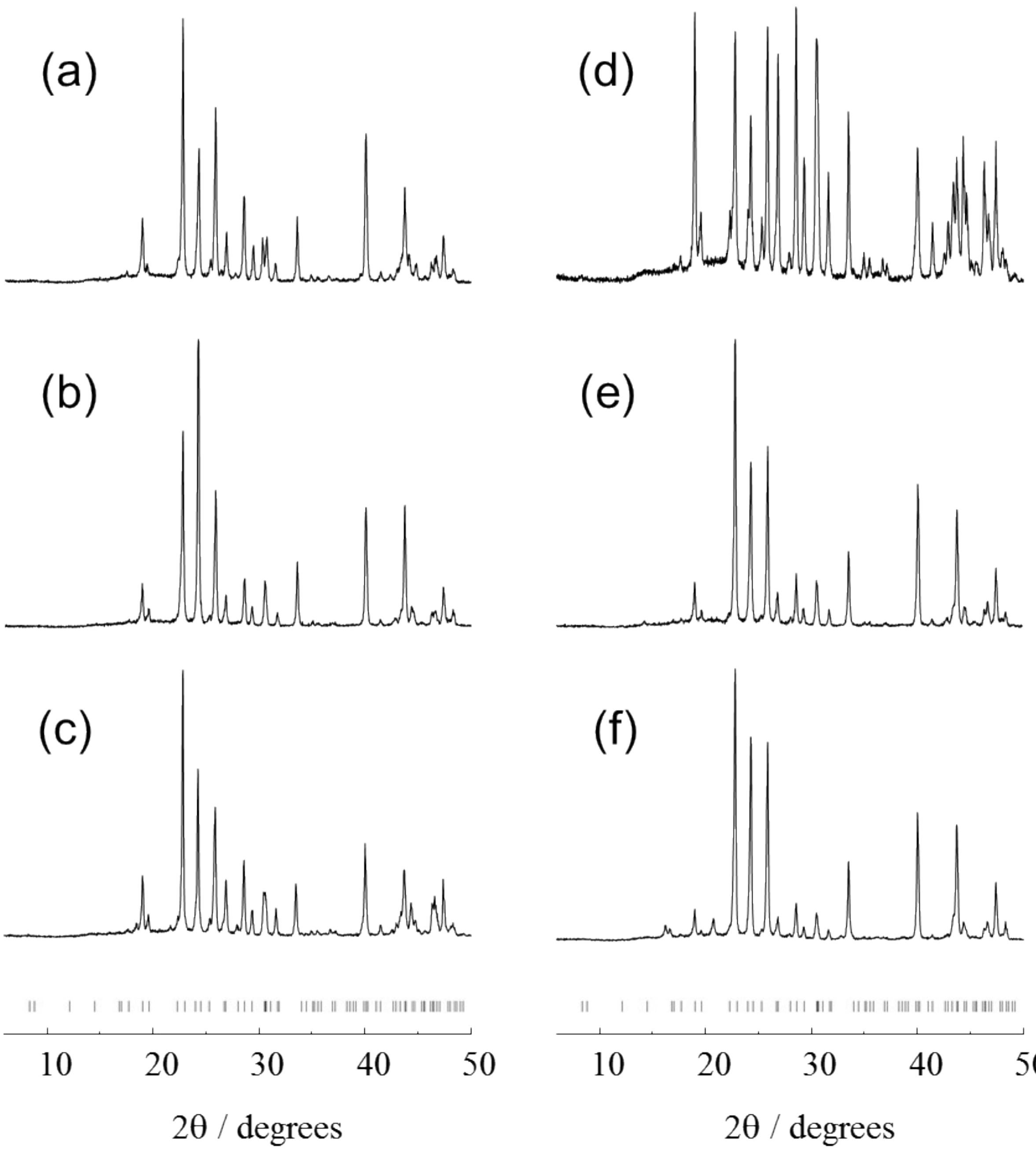

Fig. S1. X-ray diffraction patterns of the structure H hydrates at 93 K: (a) CH_4 + 2,2-dimethybutane hydrate; (b) CH_4 + 2,3-dimethybutane hydrate; (c) CH_4 + 3-methypentane hydrate; (d) CH_4 + 2-methylbutane hydrate; (e) CH_4 + methylcyclopentane hydrate; (f) CH_4 + methylcyclohexane hydrate. The curves in each pattern represent the observed intensities. The line marks below each pattern show the calculated peak positions for hexagonal ice.

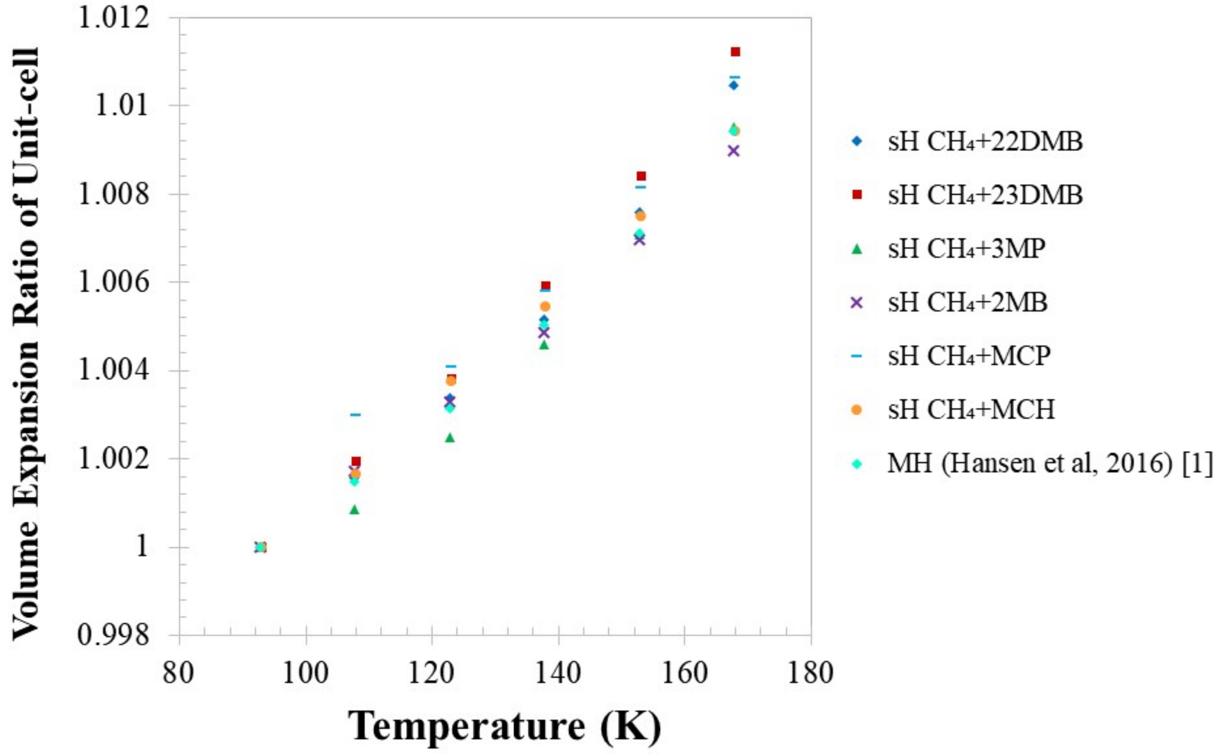

Fig. S2. Temperature effect on volume expansion ratio of unit-cell for six types sH hydrates and sI CH_4 hydrate (MH).[1]

Reference

[1] Hansen, T. C.; Falenty, A.; Kuhs, W. F. Lattice Constants and Expansivities of Gas Hydrates from 10 K up to the Stability Limit. *J. Chem. Phys.* **2016**, 115**,** 7066–7070.
